# Comparison of Two Diagnostic Methods for the Detection of Hepatitis B Virus Genotypes in the Slovak Republic

**DOI:** 10.3390/pathogens11010020

**Published:** 2021-12-24

**Authors:** Mariia Logoida, Pavol Kristian, Andrea Schreiberova, Patrícia Denisa Lenártová, Veronika Bednárová, Elena Hatalová, Ivana Hockicková, Sylvia Dražilová, Peter Jarčuška, Martin Janičko, Štefan Porhinčák, Monika Halánová

**Affiliations:** 1 Department of Epidemiology, Faculty of Medicine, Pavol Jozef Šafárik University, 040 11 Kosice, Slovakia; mariia.logoida@student.upjs.sk (M.L.); veronika.bednarova@upjs.sk (V.B.); elena.hatalova@upjs.sk (E.H.); monika.halanova@upjs.sk (M.H.); 2 Department of Infectology and Travel Medicine, Faculty of Medicine, Louis Pasteur University Hospital, Pavol Jozef Šafárik University, 041 90 Kosice, Slovakia; patricia.denisa.lenartova@upjs.sk (P.D.L.); ivana.hockickova@upjs.sk (I.H.); 3 Department of Epizootiology, Parasitology and Protection of One Health, University of Veterinary Medicine and Pharmacy, 041 81 Kosice, Slovakia; andrea.schreiberova@uvlf.sk; 4 2nd Department of Internal Medicine, Faculty of Medicine, Louis Pasteur University Hospital, Pavol Jozef Šafárik University, 040 11 Kosice, Slovakia; sylvia.drazilova@upjs.sk (S.D.); peter.jarcuska@upjs.sk (P.J.); martin.janicko@upjs.sk (M.J.); 5 Centre of Applied Computer Science, P. J. Safarik University, 040 01 Kosice, Slovakia; stefan.porhincak@upjs.sk

**Keywords:** hepatitis B virus, HBV genotype, genotyping methods, sequencing

## Abstract

The hepatitis B virus (HBV), belonging to the *Hepadnaviridae* family, is responsible for a global health concern still in the 21st century. The virus is divided into 10 genotypes, which differ in geographical distribution and in their effect on disease progression and transmission, susceptibility to mutations, and response to treatment. There are many methods for diagnostics of HBV and differentiating its genotypes. Various commercial kits based on real-time polymerase chain reaction (RT PCR) and hybridization available, as well as whole genome sequencing or the sequencing of only individual parts of the genomes. We compared a commercial kit AmpliSens HBV-genotype-FRT, based on RT PCR, with an adapted method of amplification of the surface genomic region combined with Sanger sequencing. In the examined samples we identified the A, B, C, D, and E genotypes. By PCR with Sanger sequencing, the genotypes were determined in all 103 samples, while by using the commercial kit we successfully genotyped only 95 samples, including combined genotypes, which we could not detect by sequencing.

## 1. Introduction

Viral hepatitis B is one of the most common viral infections in humans; it is spread worldwide and represents a global public health problem. The prevalence of hepatitis B varies worldwide and ranges from 0.7% of those infected in the adult population in low endemic regions to 6.2% in high endemic regions. In 2010, according to a European Centre for Disease Prevention and Control (ECDC) Technical report, the Slovak Republic was classified as a low endemic country for HBV infection [1]. However, there are still groups of the population in which the prevalence is higher. The cross-sectional population-based Hepa-Meta study, focused on the prevalence of viral hepatis, metabolic syndrome, and selected bacterial and parasitic infectious diseases in the Roma population living in segregated settlements, detected a 12.5% prevalence of HBV surface antigen (HBsAg) in these citizens [2,3]. Between years 2015 and 2020, in Slovak Republic there were on average 144 cases reported per year [4,5].

HBV is a partially double-stranded DNA virus roughly 3200 nucleotides in length and belonging to the *Hepadnaviridae* family [6]. The genome contains four partially or entirely overlapping open reading frames (C, P, S, and X), which encode seven proteins: pre-core and core protein (HBeAg and HBcAg); polymerase protein (reverse transcriptase, RT), preS1, preS2 and small hepatitis B surface proteins (SHB) (three forms of HBsAg) and X protein (transcriptional trans-activator protein) [7,8].

HBV is currently classified into 10 genotypes, A-J, according to differences in the complete genomic sequence, with the difference between the individual genotypes being about 8% and between subgenotypes about 4% [9,10]. The estimated mutation rate in HBV is determined to be approximately 10 ^−4^ to 10 ^−6^ nucleotide substitutions/site per year [11,12,13]. Significant differences between genotypes in geographical distribution have been observed. HBV genotype A is more common in Europe (A2), North America (A2), and Africa (A1, A3–A6), while genotype B and C is the most prevalent in Asia and the Pacific. HBV genotype D was detected in Middle East (D1), Central Asia (D1), Europe (D2), Japan (D2), Australia and Oceania (D4), India (D5), and South Africa (D6), and subgenotype D3 is spread worldwide. Genotype E is typical for Africa, while genotypes F and H for South, Central, and North America. HBV genotype G was detected in the USA, Germany, Italy, the UK, and France, and genotype I has been found in Asia and genotype J in Japan [7,14]. There is also a link between genotypes and their modes of transmission. In addition, different HBV genotypes can have variability in their clinical outcomes and response to treatment, including the development of drug resistance [15,16,17]. Therefore, data on the genotypes circulating in the population help to detect transmission pathways and serve as an epidemiological tool for monitoring the mode of transmission and clustering of the virus [18].

A lot of different techniques are available for HBV genotyping. Sequence and phylogenetic analysis of the entire HBV genome is still considered the gold standard for genotyping [19]. However, full genomic sequencing appears to be ineffective for regular use in clinical practice, particularly due to high costs, the time consumed, and the expertise required. Sequencing and phylogenetic analysis can be performed only on parts of the genome; most often the S region (S-surface) is used for genotyping. In general, the HBV S gene sequence is enough to assign genotypes [8,20]. In addition, to determine the HBV genotype, other methods can be used, such as restriction fragment length polymorphisms (RFLP), [21], PCR with specific primers and probes for single genotypes [22,23] or other methods based on hybridization technologies [14,24].

In this study, we compared the commercial kit AmpliSens HBV-genotype-FRT, based on real-time PCR using specific primers and probes for the A, B, C, and D genotypes with adapted direct and nested PCR, with primers which are used in INNO-LiPA HBV Genotyping kits. These primers target the HBV surface genomic region, which overlaps with the polymerase region, and this also allows us to detect the possible presence of an escape or resistance mutation [25]. The aim of this study was to compare two different diagnostic methods for the detection of Hepatitis B virus genotypes to better understand the molecular epidemiology of HBV in Slovakia, which can lead to better understanding of the origins and distribution patterns of HBV genotypes in patients in Slovakia, thus ensuring better patient management and appropriate treatment.

## 2. Results

A total of 103 people positively diagnosed with viral hepatitis B were examined for the detection of HBV genotype.

From the 103 examined serum samples, 95 were successfully genotyped by real-time PCR using the commercial kit AmpliSens HBV-genotype-FRT. In all 103 samples, the genotypes were determined by amplification of the surface genomic region (using primers from the INNO-LiPA assays) and Sanger sequencing.

**Figure 1:**
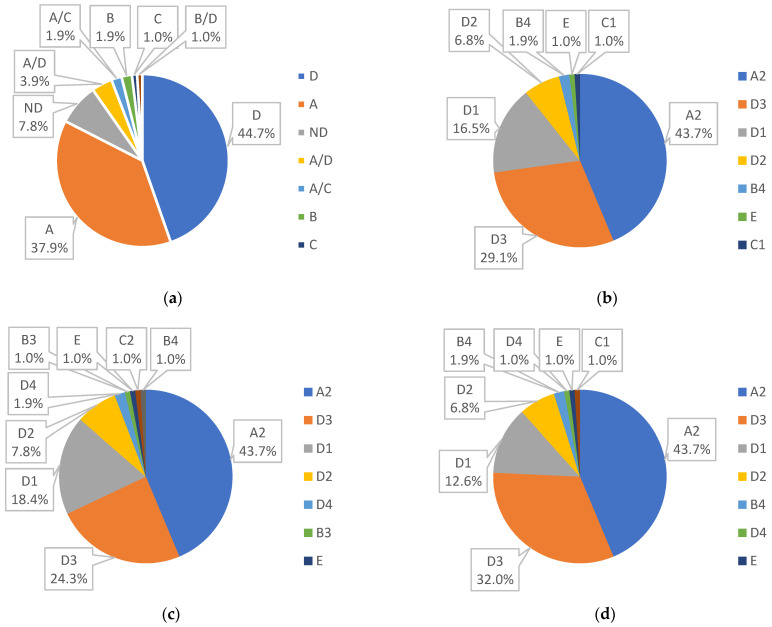
(**a**) Genotyping by RT PCR; (**b**) genotyping by NCBI annotation; (**c**) genotyping by the Geno2pheno[hbv] tool; (**d**) genotyping by the MEGA X software Phylogenetic tree.

Among all the samples successfully analyzed by AmpliSens HBV-genotype-FRT commercial kit, the prevalent genotype was D with 44.7%; genotype A was second with 37.9%, and the most prevalent combination genotype was A/D, represented by 3.9%. Other genotypes had 1% to 1.9% occurrence, as shown in Figure 1a.

The AmpliSens kit could not detect viral DNA in 8 of our samples (7.8%), which were determined by amplification of the surface genomic region and Sanger sequencing. Of these 8 samples, 5 were determined as a genotype D and 2 as genotype A. One of these 8 samples was determined as genotype E, as the AmpliSens kit is limited only to genotypes A, B, C, and D (Appendix A Table A1).

By amplification of the surface genomic region (using primers from the INNO-LiPA assays) and Sanger sequencing, we determined the genotype in all 103 samples. Obtained sequences were analyzed by tools, NCBI, Geno2Pheno, and Phylogenetic analysis by Mega X Software. According to NCBI annotation, we found the most prevalent genotype was genotype D, with subgenotype D1—16.5%, D2—6.8%, D3—29.1%, followed by A2—43.7%, and genotypes C1, E, and B4, each with 1% or 1.9%, as show in Figure 1b. Genotyping by Geno2Pheno[hbv] tools provided us the similar results: The most prevalent genotype was D (D1—18.4%. D2—7.8%, D3—24.3%, D4—1.9%), next A2—43.7% and genotypes B3, B4, and C2, E, each with 1%, Figure 1c.

By phylogenetic analysis, we verified our genotypes and subgenotypes and detected genotypes D with 52.4% (D3 with 32%, D1 with 12.6%, D2 with 6.8%, and D4 with 1%), A2 with 43.7%, B4 with 1.9%, and C1 and E each with 1%, as shown in Figure 1d.

We discovered that all results of genotyping produced by different tools were the same on the genotype level, with differences only at the subgenotype level. The results of phylogenetic analysis were very similar to the results by manual annotation in the NCBI database, with difference only in 4 samples. Three of samples were determined by NCBI annotation and by Geno2Pheno[hbv] tools as subgenotype D1, but according to phylogenetic analysis they belonged to genotype D3.

**Figure 2:**
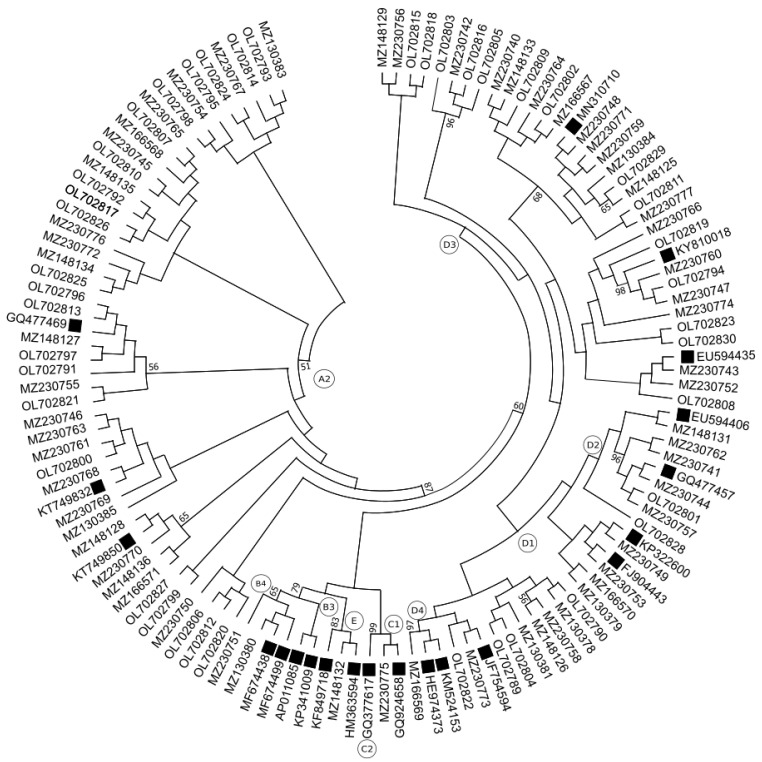
Phylogenetic analysis of the HBV S-gene region sequences. The 103 specimens were aligned with 21 representative sequences of 5 genotype (including the relevant subgenotypes) available from GenBank. Reference sequences were marked by filled square labels. The final length was 337 bp. The alignment was analyzed using the Maximum Likelihood method and Kimura 2-parameter model with 1000 bootstrap replicates in the MEGA X software. Branch nodes with bootstrap values >50 are included next to the corresponding node.

One of these samples, which was determined by NCBI annotation as a subgenotype D1, was determined by Geno2Pheno[hbv] tools as subgenotype D4 and according to phylogenetic analysis belonged to genotype D4. Nevertheless, branches with reference sequences for subgenotype D4 were arranged as a subtree for the D1 branch (Figure 2). Such ambiguities in branching could be caused by relatively short and maybe insufficiently divergent sequences.

With commercial kit AmpliSens HBV-genotype-FRT, it was possible to detect the same genotypes as with other methods (plus mixed genotypes), except for 8 samples, in which we were unable to determine genotype with this kit (Appendix A Table A1). Thus, the success rate of the commercial kit compared to other methods was 92.23%. As for the comparison of, for example, manual annotation in the NCBI database and the Geno2Pheno[hbv] tools concurred on 89.32% (on the subgenotype levels), and results of phylogenetic analysis using MEGA X software matched with the Geno2Pheno[hbv] tools in 88.35% and the results of NCBI annotation were same as analysis using MEGA X in 94.17% of samples.

**Figure 3:**
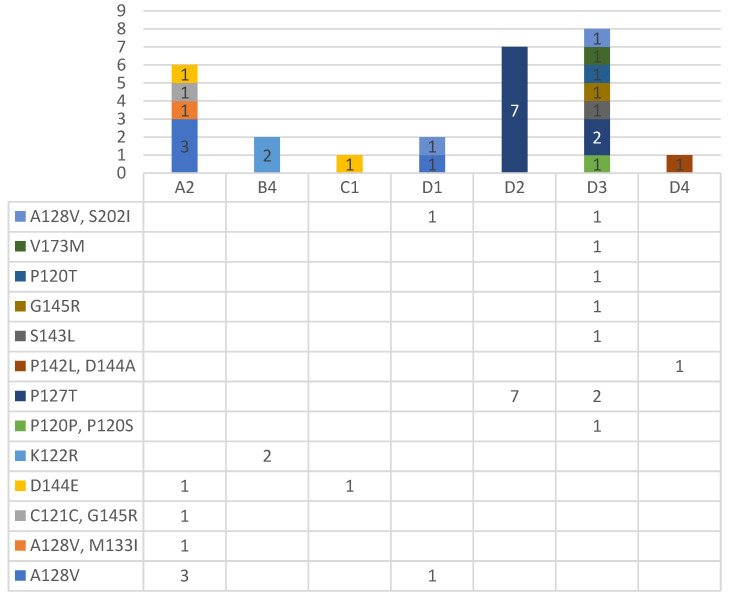
Occurrence of resistance and escape mutation divided per genotype (genotypes are marked by MEGA X phylogenetic analysis).

In addition, the obtained sequences were used for the detection of clinically important resistant and escape mutations through different online tools, such as the Geno2Pheno[hbv] tool (https://hbv.geno2pheno.org/; accessed on 5 December 2021) and the HBV-Resistance interpretation tool (http://www.hiv-grade.de/hbv_grade/deployed/grade; accessed on 5 December 2021) (Appendix A Table A2). The most common mutations were the HBsAg escape mutations—A128V and P127T, which were typical for genotypes D and A, followed by the HBsAg escape mutations—D144 E and K122R and the compensatory mutation—S202I, which can cause to resistance to Entecavir, Baraclude^®^. Other HBsAg escape mutations occurred only individually: V173M, P120T, S143L, G145R, P120P together with P120S, C121C together with G145R, A128V together with M133I, and P142L together with D144A, as shown in Figure 3. All the important mutations were detected by both instruments, except the P127T mutation, which was detected only by the HBV-Resistance interpretation tool. Regarding neutral mutations, some were detected by only one of two software. All mutation data should be interpreted with caution since it is only a prediction and the region of interest of the genes is relatively short.

## 3. Discussion

Knowledge of the circulating genotypes in a community and data on existing mutations can lead to a better understanding of the molecular epidemiology of the hepatitis B virus and contribute to better patient management and treatment. HBV genotype can be confirmed by a variety of methods, such as sequence analysis of partial or whole genome, genotype-specific PCR assays, real-time PCR, RFLP, microarray (DNAChip), or fluorescence polarization assay [26]. The reference point for all methods is sequencing and phylogenetic analysis. While whole genome sequencing is the gold standard and the most reliable method, it is cumbersome to use in large scale studies and is expensive and requires expertise [19]; thus, sequencing of only a part of the genome can be a viable alternative. Phylogenetic analysis allows relative and evolutionary relatedness of sequences to be assessed and can also be performed on individual genes, especially on S gene [14].

Commercial kits make diagnostics easier, faster, and more convenient for the routine. One of the commercial methods to genotype HBV is the INNO-LIPA^®^ HBV Genotyping (Fujirebio Europe, Tokyo, Japan) based on reverse hybridization. This method allows the identification of HBV genotypes A to G and shows high sensitivity [27], but is relatively expensive. The other available commercial kit is AmpliSens^®^ HBV-genotype-FRT PCR kit (Federal Budget Institute of Science “Central Research Institute for Epidemiology”, Moscow, Russia), based on real-time PCR with specific hybridization probes. This kit used on qualitative detection and differentiation of hepatitis B virus (HBV) genotypes A, B, C, and D.

We compared a commercial kit AmpliSens HBV-genotype-FRT, based on RT PCR, with an adapted PCR method (using primers from INNO-LiPA assay), combined with Sanger sequencing (Appendix A Table A1). By commercial kits AmpliSens HBV-genotype-FRT we determined genotype in 95 from 103 samples, which represents 92.2% of samples. This method for genotyping appears to be useful for the rapid genotyping of HBV, as is quick and easy for preparing. In addition, genotyping using the commercial kit AmpliSens HBV-genotype-FRT allows us to detect combined genotypes. But this method is limited only to genotypes A, B, C, and D. Furthermore, a disadvantage is that the single nucleotide polymorphisms (SNP) at the primer site can affect the sensitivity of method [19].

Using the PCR method adapted by us (using primers from INNO-LiPA assay) combined with Sanger sequencing, we successfully determined genotype in all 103 samples. This method appears to be very sensitive, and it allows for further increase of sensitivity with nested PCR. PCR with sequencing also opens many possibilities, as well as sequence comparison in various databases or the use of various online tools, phylogenetic analysis, and in the case of a sequence that is coding polymerase and S protein, we also have the possibility of detecting important resistant or escape mutations. This method is cheaper than commercial methods but takes more time and is technically demanding and requires expertise with processing data. Another disadvantage of sequencing using a single region of the HBV genome is the inability to determine combined genotypes [8]. We also tried to detect not only the genotype but the subgenotype, too. To obtain subgenotype, we compared our sequencing to the reference sequences using the bioinformatics tool Blast from the NCBI database. These results were compared with results obtained by Geno2Pheno[hbv] tools (https://hbv.geno2pheno.org/; accessed on 5 December 2021) and with results from phylogenetic analysis by the MEGA X software. Only 14 samples have a different subgenotype using one of the methods, but they have the same results according to at least two other methods. All the other samples have the same subgenotype according to different methods. Overall, on the subgenotype level, the results of NCBI annotation, Geno2Pheno[hbv] tools and phylogenetic analysis using the MEGA X software matched in 86.41% of the samples, and these methods showed 80.58% consistency with RT PCR. However, the fact that this study is limited by the short lengths of gene sequences must be taken into consideration.

In addition, the obtained sequences were used for the detection of clinically important resistant and escape mutations through online tools Geno2Pheno[hbv] (https://hbv.geno2pheno.org/; accessed on 5 December 2021) and the HBV-Resistance interpretation tool (http://www.hiv-grade.de/hbv_grade/deployed/grade; accessed on 5 December 2021). From all 103 samples, the important clinical mutation was detected in 27 samples, which represents 26.21%. The most prevalent was the HBsAg escape mutations A128V and P127T, followed by the HBsAg escape mutations D144 E and K122R and the compensatory mutation S202I. Other mutations occurred individually, such as P120T, S143L, and G145R, while mutation M133I was together with A128V and P142L was together with D144A (Appendix A Table A2). The HBsAg vaccine escape mutation A128V was associated with occult HBV infection [28]. This is the most typical mutation for genotype D, but we found this mutation in genotype A, too. Of interest was the fact that two samples that had resistant mutation S202I also had the A128V escape mutation. Compensatory mutation S202I, which can cause resistance to Entecavir and Baraclude^®^, was among the most described mutations in the RT region [29,30]. We detected this mutation only in genotype D and only together with the A128V escape mutation. A mutation which affects the 144 or 145 amino acid position can be responsible for vaccine escape and failure of immunoglobulin (IG) therapy and detection [31,32]. Liver transplant patients infected with these escape mutations were described as having a worse clinical outcome compared to other patients [33]. Mutation P120T is also responsible for vaccine, therapy (IG) and detection failure [32,34]. The S143L immune escape mutation was also previously described in genotype D [35,36]. There is not sufficient data available on the K122R and P127T mutations to interpret these mutations, but they were also detected in other studies [29,37,38,39], though mutation K122R was detected only in genotype B. Mutation P127T could not be detected by the Geno2Pheno[hbv] tools as HBsAg escape; this variant was detected only by the HBV-resistance interpretation tool.

**Figure 4:**
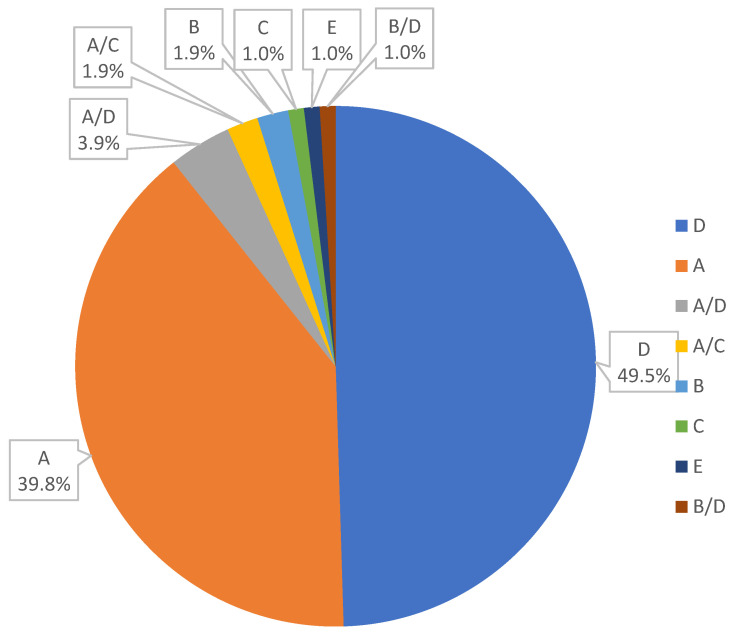
Prevalence of the HBV genotypes among patients in the Slovak Republic.

To our knowledge, this is the first published study based on data from HBV genotyping in the Slovak Republic. We compared two diagnostic methods for the detection of Hepatitis B Virus genotypes in the Slovak Republic, commercial kit AmpliSens HBV-genotype-FRT versus an adapted PCR method combine with Sanger sequencing. Both methods have advantages and disadvantages: Genotyping by commercial kit is quicker, and able to detect combined genotypes, while genotyping by PCR with Sanger sequencing appears to be a more informative and sensitive. The optimal approach could be first using the commercial kit for faster routine diagnostics and secondly, in case of ambiguous or unspecified results verify these results by sequencing and phylogenetic analysis. In addition, sequencing can be useful for following some clinically important mutations and prediction of response to treatment. We found that the most common genotype was D (49.5%), followed by genotype A (39.8%), genotype B with 1.9%, and genotypes C and E with 1%. We also detected a prevalence of the genotype combination A/D, represented by 3.9%, followed by the combination A/C and B/D, with 1.9% and 1% prevalence respectively (Figure 4).

Obtaining prevalence of HBV in Slovak Republic gives us an opportunity to compare our data with data from neighboring states. In the Czech Republic, for example, a prevalence of genotype A (*n* = 33; 73% and 67.1%) over D (*n* = 12; 27% and 28.4%) was described [40,41], and genotypes B and C (3.4% and 1.1%, respectively) were also found [41]. In Poland, the most common genotype determined was genotype A with 67%, followed by genotype D (20%), and genotype H (5%) and mixed A/D (5%). In addition, genotype F, combined genotypes D/G, A/C, and D/F, individually, were found in Poland [42]. In Ukraine, genotype D (52.4%), followed by A (14.2%) and C (4.7%), was the most prevalent [43]. In a report of the prevalence of HBV genotypes in Central and Eastern Europe as a whole, the prevalence of genotype D was 48% and genotype A was 42%, and only a few cases of genotype B, C, E, and F were detected [44]. According to that study, genotype A predominated in Poland (77%) and the Czech Republic (67%), as compared to Hungary (47%), Lithuania (41%), Croatia (8%) and Germany (32%), and genotype D was the most common for Lithuania (54%), Germany (58%), Romania (67%), Croatia (80%), and Russia (93%). About 8% of this European cohort’s patient had a mixed genotype mutation. Most of them were in Romania, where 27% of the samples proved to have more than one genotype and 82% of the combination genotypes took the form of the A/D genotype combination. Our results are comparable to the prevalence of HBV in neighboring countries.

## 4. Materials and Methods

### 4.1. Population Study

Between June 2019–October 2021, a total 103 serum samples were collected from HBsAg positive patients. To isolate serum from patients, a sample of approximately 5–7 mL of whole blood was collected into EDTA vacutainers. Serum was stored at −80 °C until testing. All patients were informed and provided written consent prior to examination. This study was approved by Ethics Committee of the L. Pasteur University Teaching Hospital, No. 2019/EK/4022.

### 4.2. DNA Isolation

HBV DNA was isolated from 400 μL of serum using the QIAamp^®^ DNA Mini kit (QIAGEN GmbH, Hilden, Germany) in accordance with the manufacturer’s protocol and dissolved in 40 μL of elution buffer. Strict precaution was taken to prevent contamination. Subsequently, the DNA thus isolated was used for amplification and, if necessary, stored at −20 °C (for a short time) or at −80 °C (for a long time) for further use.

### 4.3. HBV Genotyping by Real-Time PCR

HBV genotyping was done using 10 μL of extracted DNA and the commercial kit AmpliSens^®^ HBV-genotype-FRT (AmpliSens, Federal Budget Institute of Science “Central Research Institute for Epidemiology”, Moscow, Russia). The AmpliSens^®^ HBV-genotype-FRT PCR kit is a nucleic acid amplification test for qualitative detection and differentiation of HBV genotypes A, B, C, and D. Amplification was performed on a LightCycler ^®^ 480 Real-Time PCR System (ROCHE Diagnostics, Mannheim, Germany).

### 4.4. HBV Amplification by PCR (Direct or Nested)

For PCR amplification, we adapted specific genotyping primers for the surface genomic region from INNO-LiPA HBV genotyping assay (Fujirebio US, Inc., Malvern, PA, USA), [24]. PCR amplification was performed using the primers: HBPr134 (5′-TGCTGCTATGCCTCATCTTC-3′) and HBPr135 (5′-CARAGACARAA-GAAAATTGG-3′) for direct PCR, HBPr75 (5′-CAAGGTATGTTGCCCGTTTGTCC-3′) and HBPr94 (5′-GGYAWAAAGGGACTCAMGATG-3′) for nested PCR, and 5 x HOT FIREPol^®^ Blend Master Mix Ready to Load (Solis BioDyne, Tartu, Estonia) and 5 μL of DNA samples were also used for direct and 2 μL (products of the direct round PCR) for nested PCR. Amplification was performed in a standard thermocycler (T1Thermocycler, Biometra GmbH, Göttingen, Germany), and the thermal cycling parameters used were: initial denaturation −95 °C for 12 min and 30 cycles of 95 °C for 20 s, 52 °C for 40 s, 72 °C for 60 s, and a final elongation of 72 °C for 10 min. The thermal cycling parameters were the same for both the direct and nested PCR. PCR products were visualized using 1% agarose gel electrophoresis. Nested PCR was performed only if direct PCR was not sufficient. Generally, the first round of PCR was sufficient, and the nested (second round) PCR was needed only five time. We obtained 409 bp products from direct and 341 bp from nested PCR.

### 4.5. Determination of HBV Genotype and Phylogenetic Analysis

Amplicons were sent for Sanger sequencing (Microsynth AG, Wien, Austria). The obtained chromatograms were analyzed and edited using the MEGA X software [25]. If the sequences had a short length or poor sequence quality, those samples were sent for Sanger sequencing repeatedly. All sequences were assembled in GeneTool Lite 1.0 software (BioTools Inc., Edmonton, AB, Canada). The sequences were compared to the reference sequences using the bioinformatics tool Blast from the U.S. National Centre for Biotechnology Information (NCBI, Bethesda, MD, USA) (http://www.ncbi.nlm.nih.gov/; accessed on 5 December 2021). In addition, these sequences are deposited in the NCBI GenBank (Accession numbers: MZ130378-MZ130381, MZ130383-MZ130385, MZ148125-MZ148129, MZ148131-MZ148136, MZ166567-MZ166571, MZ230740-MZ230777, OL702789-OL702830). The samples were genotyped by annotation in the NCBI database to detect genotype and subgenotype, which we compared with results using the Geno2Pheno[hbv] tools from the Max-Planck Institute for Informatics (https://hbv.geno2pheno.org/; accessed on 5 December 2021). As our sequencing regions overlapped the surface and polymerase genes, this also allowed us to check our sequences for the presence of resistance and escape mutations. For this, we used the same Geno2Pheno[hbv] tools and compared the results with the HBV-Resistance interpretation tool algorithm available online and based on the Stanford HIValg Software (http://www.hiv-grade.de/hbv_grade/deployed/grade; accessed on 5 December 2021). The genotyping results were also confirmed by phylogenetic analysis using the Mega X software [45]. A phylogenetic tree was constructed using a Maximum likelihood tree with Kimura-2-parameter substitution methods, and bootstrap values were calculated from 1000 replicates. The gene bank reference sequences of the major genotypes and subgenotypes that were included in phylogenetic analysis are: KP322600, FJ904443, JF754594, EU594406, GQ477457, MN310710, EU594435, KY810018, KM524153, HE974373, KT749850, KT749832, GQ477469, MF674438, MF674499, KP341009, AP011085, GQ924658, GQ377617, KF849718, HM363594.

## Data Availability

The data presented in this study are available on request from the corresponding author.

## Appendix A

**Table A1:** HBV genotyping, comparison of different methods.

Sample	Accession Numbers	Real-Time PCR	NCBI Annotation	Genotype by hbv.geno2pheno	MEGA X Software Phylogenetic Tree
1001	MZ130378	D	D1	D1	D1
1002	MZ130379	D	D1	D1	D1
1003	MZ130380	B	B4	B4	B4
1004	MZ130381	B/D	D1	D1	D1
1005	MZ130383	A	A2	A2	A2
1006	MZ130384	D	D3	D1	D3
1007	MZ130385	A	A2	A2	A2
1008	MZ148125	D	D3	D3	D3
1009	MZ148126	D	D1	D1	D1
1010	MZ148127	A/C	A2	A2	A2
1011	MZ148128	A	A2	A2	A2
1012	MZ148129	D	D3	D3	D3
1014	MZ148131	D	D2	D2	D2
1015	OL702789	D	D1	D1	D1
1016	MZ148132	-	E	E	E
1017	MZ148133	D	D3	D3	D3
1018	MZ148134	A	A2	A2	A2
1019	MZ148135	A	A2	A2	A2
1020	MZ148136	A	A2	A2	A2
1021	MZ166567	D	D3	D3	D3
1022	MZ166568	A	A2	A2	A2
1023	MZ166569	D	D1	D4	D4
1024	MZ166570	D	D1	D1	D1
1025	MZ166571	A	A2	A2	A2
1026	MZ230740	D	D3	D3	D3
1027	MZ230741	A/D	D2	D2	D2
1028	MZ230742	D	D3	D3	D3
1029	MZ230743	D	D3	D1	D3
1030	MZ230744	D	D2	D2	D2
1031	MZ230745	A	A2	A2	A2
1032	MZ230746	A	A2	A2	A2
1033	MZ230747	-	D3	D3	D3
1034	MZ230748	D	D3	D3	D3
1035	MZ230749	D	D1	D1	D1
1036	MZ230750	A	A2	A2	A2
1037	MZ230751	B	B4	B3	B4
1039	MZ230752	D	D3	D1	D3
1040	MZ230753	D	D1	D1	D1
1041	MZ230754	A/D	A2	A2	A2
1042	MZ230755	A	A2	A2	A2
1043	MZ230756	D	D3	D3	D3
1044	MZ230757	D	D2	D2	D2
1045	MZ230758	A/D	D1	D1	D1
1046	MZ230759	D	D3	D3	D3
1047	MZ230760	D	D3	D3	D3
1048	OL702827	-	A2	A2	A2
1049	MZ230761	A	A2	A2	A2
1050	MZ230762	D	D2	D2	D2
1052	MZ230763	A	A2	A2	A2
1053	MZ230764	D	D3	D3	D3
1054	MZ230765	A	A2	A2	A2
1055	MZ230766	D	D3	D3	D3
1056	MZ230767	A	A2	A2	A2
1057	MZ230768	A	A2	A2	A2
1058	MZ230769	A	A2	A2	A2
1059	MZ230770	A	A2	A2	A2
1060	MZ230771	D	D3	D3	D3
1061	MZ230772	A	A2	A2	A2
1063	MZ230773	D	D1	D1	D1
1064	MZ230774	D	D3	D1	D3
1065	MZ230775	C	C1	C2	C1
1066	MZ230776	A/D	A2	A2	A2
1067	MZ230777	-	D3	D3	D3
1068	OL702790	D	D1	D1	D1
1070	OL702791	A	A2	A2	A2
1071	OL702792	A	A2	A2	A2
1072	OL702793	A	A2	A2	A2
1073	OL702794	D	D3	D3	D3
1074	OL702795	C/A	A2	A2	A2
1075	OL702796	A	A2	A2	A2
1076	OL702797	A	A2	A2	A2
1077	OL702798	A	A2	A2	A2
1078	OL702799	A	A2	A2	A2
1079	OL702800	A	A2	A2	A2
1080	OL702828	D	D1	D2	D2
1081	OL702801	D	D2	D2	D2
1082	OL702802	D	D3	D3	D3
1083	OL702803	D	D3	D3	D3
1084	OL702804	-	D1	D4	D1
1085	OL702805	D	D1	D1	D3
1086	OL702806	A	A2	A2	A2
1087	OL702807	A	A2	A2	A2
1088	OL702808	-	D3	D3	D3
1089	OL702809	D	D3	D3	D3
1090	OL702810	-	A2	A2	A2
1091	OL702811	D	D3	D3	D3
1092	OL702812	A	A2	A2	A2
1093	OL702813	A	A2	A2	A2
1094	OL702814	A	A2	A2	A2
1095	OL702815	D	D1	D1	D3
1096	OL702816	D	D3	D3	D3
1097	OL702817	A	A2	A2	A2
1098	OL702818	D	D2	D2	D3
1099	OL702819	D	D3	D3	D3
1100	OL702820	A	A2	A2	A2
1101	OL702821	A	A2	A2	A2
1103	OL702822	D	D1	D1	D1
1104	OL702829	D	D3	D3	D3
1105	OL702830	D	D3	D1	D3
2001	OL702823	D	D3	D3	D3
2002	OL702824	A	A2	A2	A2
2003	OL702825	A	A2	A2	A2
2005	OL702826	A	A2	A2	A2

**Table A2:** Detection of resistance and escape mutations.

Sample	Mutations RT Domain	Drug Resistance Mutation	Mutations SHB Protein	Escape Mutations SHB Protein
1001	G127R, M129L, N131D, Y135S, Q215S		T127P, T189I, S207R	
1002	Y135S		T127P	
1003	N124H, N134D, K149Q, V207M		K122R, M198I, F200Y	K122R
1004	G127R, Y135S		T127P	
1005	V207I, L217R		M198I, W199L, L209V	
1006	Q130P, Y135S		T127P	
1007	W153GRW, L217R, S219A		L209V, D144DE, S210R	D144E-Vaccine, Therapy (IG), Detection
1008	F122L, Q130P, Y135S, F221Y		T127P, L213I	
1009	Y135S, S202IS	202I-compensatory mutation, possible resistance to Entecavir, Baraclude^®^	T127P, A128AV, T189I, V194FILV	A128V-Vaccine
1010	L217R, S219A		L209V, S210R	
1011	L217R		L209V	
1012	F122L, T128INST, Q130P, Y135S		P120PS, T127P, S207N	P120P-Vaccine P120S-Vaccine, Detection
1014	F122V, H126R, Q149K		T118A, P127T	P127T
1015	G127GR, M129L, Y135S, Q149KQ		T127P	
1016			T189I	
1017	F122L, Q130P, Y135S, Q215H		T127P, S207T	
1018	S159T, L217R		L209V, L216	
1019	W153R, L217R		S207N, L209V	
1020	R120G, N124H, L217R		L209V	
1021	F122L, Q130P, Y135S		T127P, V177A, Y206C	
1022			A128AV, S207N, V209L	A128V-Vaccine
1023	H126R, M129L, Y135S, Q149K, Q215P		T118A, T127P, P142L, D144A, S204N, S207R	P142L, D144A-Vaccine, Therapy (IG), Detection
1024	Y135S		T127P	
1025	L217R		P188L, L209V, P211L	
1026	F122L, Q130P, Y135S, Q215H		T127P, S207T	
1027	H126R		T118A, P127T	P127T
1028	F122L, H124N, Q130P, Y135S, V190M, Q215H, S219A		T127P, S204N, S207T, I208T, S210R	
1029	F122L, Y135S		T127P	
1030	H126R		T118A, P127T	P127T
1031	L217R, S219A		S207N, L209V, S210R	
1032	L217R, S219A		L209V, S210R	
1033	N118T, F122L, Q130P, Y135S, V191L, Q215S		I110L, T127P, G159A, W182C, V190A, Y206S, S207R, P214L	
1034	F122L, Q130P, Y135S		T127P	
1035	Y135S		T127P, A128AV	A128V-Vaccine
1036	V163I, S213T, S219A		A159G, Y161F, A194V, S204K, S210R, P214L	
1037	N124H, N134D, L220I		K122R, V168A, F200Y	
1039	F122L, Y135S, S202IS	202I-compensatory mutation, possible resistance to Entecavir, Baraclude^®^	T127P, A128AV, V194FV	A128V-Vaccine
1040	Y135S		T127P	
1041	V112A, K212T		S204R, V209L	
1042	S213T, L217R		Y161F, S204R, Y206S, L209V	
1043	F122L, Q130P, Y135S, L164M		T127P, S143L, S204N, S207N	S143L-Vaccine, Detection
1044	H126R		T118A, P127T	P127T
1045	Y135S		T127P, T189I	
1046	F122L, Q130P, Y135S		T127P	
1047	N118T, F122L, Q130P, Y135S, V191L, Q215S		I110L, T127P, G159A, W182C, V190A, Y206S, S207R, P214L	
1048	L217R		L209V	
1049	L217R, S219A		L209V, S210R	
1050	H126R		T118A, P127T	P127T
1052	L217R, S219A		L209V, S210R	
1053	F122L, Q130P, Y135S, R153QR		T127P, G145GR	G145R-Vaccine, Therapy (IG), Detection
1054	S159T		A194V, S207N, V209L	
1055	S117T, F122L, Q130P, Y135S, I187L, K212N, Q215P		T127P, S204T, Y206C, S207R	
1056	R138K, V142I		A128AV, G130S, M133I, V209L	A128V-Vaccine, M133I-Therapy (IG), Detection
1057	L217R, S219A		L209V, S210R	
1058	I187L, V190M, L217R, S219A		L209V, S210R	
1059	L217R		L209V	
1060	F122L, Q130P, Y135S		T127P	
1061	S159T, L217R		A128AV, L209V	A128V-Vaccine
1063	M129L, Y135S		T127P	
1064	F122L, T128N, Y135S, Q215P		P120T, T127P, S207R	P120T-Vaccine, Therapy (IG), Detection
1065	M129L, K149Q		D144E, V159A, A177V, S210N, I213L	D144E-Vaccine, Therapy (IG), Detection
1066	S159T, L217R, S219A		L209V, S210R	
1067	F122L, Q130P, Y135S		T127P	
1068	G127R, M129L, N131D, Y135S, Q215S		T127P, T189I, S207R	
1070	R110G, L217R		Q101R, V184A, L209V	
1071	W153R, L217R		S204N, S207NS, L209V	
1072	S106AT, V163I, L199V, V207I, L217R		S193L, A194V, M198I, W199L, L209V	
1073	N118T, F122L, Q130P, Y135S, V191L, Q215S		I110L, T127P, G159A, W182C, V190A, Y206S, S207R, P214L	
1074	M129IM, W153QR, V191IV, K212T, L217R, L220I		C121CY, G145GR, W182 *W, S204R, L209V	G145R-Vaccine, Therapy (IG), Detection; C121Y-Detection
1075	W153R, K212R, L217R, S219A		S204D, L209V, S210K	
1076	W153R, L217R, S219A		L209V, S210R	
1077	K212T, L217R		S204R, L209V	
1078	Y151FY, W153R, L179FL, L217R		T143ST, L209V	
1079	L217R, S219A		L209V, S210R	
1080	A200AV		P127T, L192FL	P127T
1081	H126R		T118A, P127T	P127T
1082	F122L, Q130P, Y135S		T127P, V177AV	
1083	F122L, Q130P, Y135S, Q215H		T127P, S207T	
1084	G127R, M129L, Y135S, Q149K, I187L, V190M		T127P, S207N	
1085	M129L, Y135S, V173M, Q215H, L217R, S219A, F221Y	V173M	T127P, S207T, L209V, S210R, L213I	
1086	L217R		A194V, Y200FY, S204NS, L209V	
1087	I121N, L217R		S113T, A194V, S207N, L209V	
1088	V103IV, F122L, Q130PQ, Y135S		T127P	
1089	R110GR, F122L, Q130P, Y135S		T127P	
1090	L217R, S219A		L94LS, F158FS, S193L, P203L, S204N, L205P, Y206F, S207N, L209V, S210R	
1091	F122L, Q130P, Y135S		T127P	
1092	L217R		A194V, S204N, L209V	
1093	W153R, L217R, S219A, L220I		S204N, S207N, L209V, S210K	
1094	V207IMV, L217LR, S219AS		M198IM, W199LW, L209LV, S210RS	
1095	Y135S		T127P, G159A, S207N	
1096	F122L, H124N, Q130PS, Y135S, V190M, Q215H, S219A		T127P, S204N, S207T, I208T, S210R	
1097	R110G, W153R, S159T, K212R, L217R, S219A, Y221F		S204D, S207N, L209V, S210R, I213L	
1098	H126R, L132LM, Y135HY, S213ST		T118A, P127T, Y200FY, S204RS, S207N, P211HP	P127T
1099	F122L, Q130P, Q215S		P127T, S136Y, S207R	P127T
1100	L217R, S219A		V96G, A194V, S204N, Y206C, L209V, S210K	
1101	L217R, S219A		Y161F, S204NS, L209V, S210R	
1103	F122V, M129L, Y135S		T127P	
1104	F122L, Q130P, Y135S, F221Y		T127P, L213I	
1105	F122L, Y135S, L199V		T127P, V184A, Y200C, Y206S	
2001	F122L, Q130P, Y135S		T127P, V184A, I208T	
2002	R138K, S219A		L98Q, A128V, G130N, Y161F, V209L, S210R	A128V-Vaccine
2003	S159T, L217R		L209V	
2005	W153R, L157M, S219A		S204N, S207N, V209L, S210R	
